# Supplementary figures and images for: Scale for students’ attitude towards AIGC feedback in english pronunciation learning: Development, validation and application
Source: PLoS One. 2025 Oct 24;20(10):e0335210. doi: 10.1371/journal.pone.0335210 (PMC12551826; doi:10.1371/journal.pone.0335210)

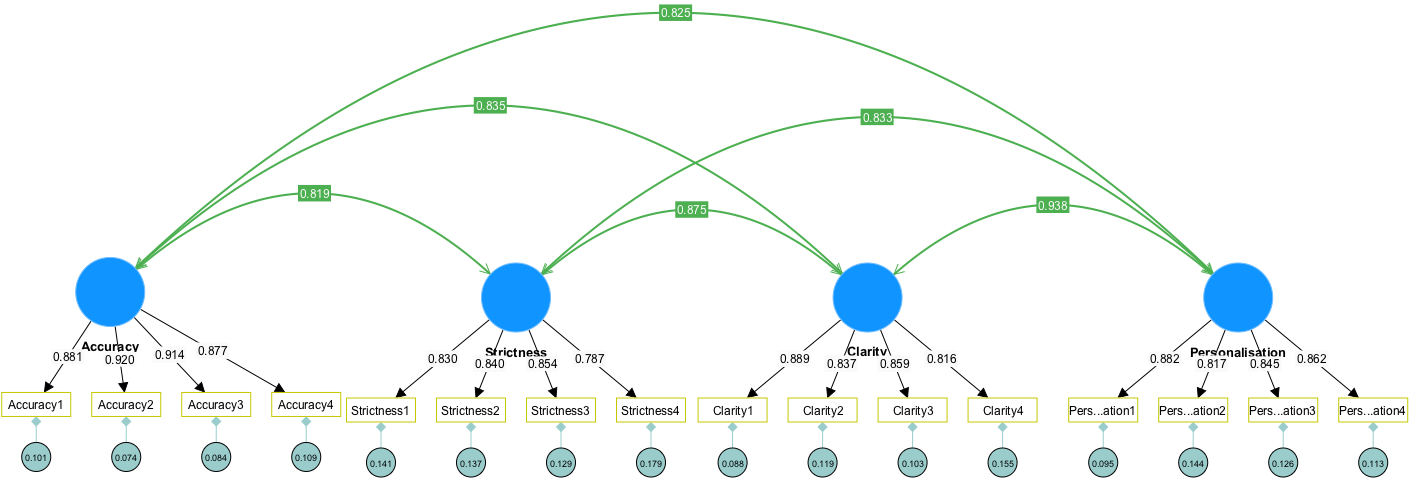

Supplement: S1 Fig — (PBG) [file pone.0335210.s003.png]
