# Supplementary material for: Scale for students’ attitude towards AIGC feedback in english pronunciation learning: Development, validation and application
Source: PLoS One. 2025 Oct 24;20(10):e0335210. doi: 10.1371/journal.pone.0335210 (PMC12551826; doi:10.1371/journal.pone.0335210)
Supplement: S2 File — (DOCX) [file pone.0335210.s004.docx]

**Appendix A: Scale for Students’ Attitude towards AIGC Feedback in English Pronunciation Learning**

**Accuracy**

A1: The generative AI software is able to accurately identify individual phoneme errors in my pronunciation (such as vowel or consonant pronunciation mistakes).

A2: The generative AI software can distinguish my errors in sentence stress and word stress, providing specific corrections.

A3: The generative AI software can correctly identify intonation errors in my speech when expressing emotions, attitudes, or intentions in different contexts and provide targeted feedback.

A4: The generative AI software can correctly detect connected speech or pause errors in my pronunciation and offer relevant feedback and suggestions.

**Strictness**

S1: Any error, no matter how small, found in my pronunciation by the generative AI software will be strictly pointed out, with suggestions for correction.

S2:If I repeat the same pronunciation error, the generative AI software will correct it strictly each time until I completely correct it.

S3:Even the smallest pronunciation errors will be rigorously corrected by the generative AI software, without any tolerance.

S4:The generative AI software takes correction very seriously, repeatedly confirming whether I have mastered the pronunciation correctly.

**Clarity**

C1: The terminology and language used by the generative AI software are clear and easy to understand, enabling me to easily follow its suggestions for improvement.

C2: The generative AI software uses simple demonstrations and examples to help me better understand its pronunciation correction suggestions.

C3: The feedback steps provided by the generative AI software are clear and well-defined, with each step being easy for me to follow.

C4: The feedback from the generative AI software is very intuitive, allowing me to understand and follow its suggestions without needing further explanation or help.

**Personalization**

P1: The generative AI software remembers my previous pronunciation issues and offers more in-depth corrections on these issues in subsequent feedback.

P2: Based on my past learning records, the generative AI software provides pronunciation practice suggestions that better meet my personal needs.

P3: The feedback provided by the generative AI software seems tailor-made for me, based on my past performance and unique pronunciation issues.

P4: The generative AI software remembers my preferences (e.g., feedback style or practice type) and reflects these preferences in future interactions.
