# Supplementary material for: Scale for students’ attitude towards AIGC feedback in english pronunciation learning: Development, validation and application
Source: PLoS One. 2025 Oct 24;20(10):e0335210. doi: 10.1371/journal.pone.0335210 (PMC12551826; doi:10.1371/journal.pone.0335210)
Supplement: Attachment — Submitted filename: Response to Reviewers comments.pdf [file pone.0335210.s006.pdf]

Dear Editor,

We sincerely thank you and the reviewers for the time and effort spent in evaluating our manuscript. We highly appreciate the constructive and insightful comments, which have helped us improve the quality and clarity of the paper. In the revised version, we have carefully addressed all the comments provided by both the academic editor and the reviewers, and we have made corresponding revisions throughout the manuscript. A point-by-point response to each comment is provided below, with explanations of the changes made and the exact locations in the revised manuscript where these changes can be found.

(1) In the Ethical Considerations section, we have added a description of the informed consent procedure. Specifically, the consent was obtained in electronic format: participants were required to read the informed consent form embedded in the online questionnaire, and by clicking the "Agree to participate" option, they indicated their consent to participate. Only after providing consent in this way could they proceed to complete the survey.

(2) To protect participants' privacy, we have removed the "major" information from the dataset and re-uploaded it as an anonymized file.

(3) We have revised the manuscript to comply with PLOS ONE guidelines regarding Response to Reviewers. Descriptive captions have been added for all files at the end of the manuscript: S1 Table, EFA dataset; S2 Table, CFA dataset; S1 File, the questionnaire. All in-text citations referring to these files have been updated to match the new captions.

(4) We thank the reviewer for pointing out the concern over discriminant validity. We

acknowledge that all participants were drawn from the same school, which may have introduced similarities in prior instruction, learning environment, and experience, thereby increasing the perceived overlap between the Personalization and Clarity dimensions. The slightly elevated HTMT value for Personalization likely reflects this sample homogeneity rather than a flaw in the scale design. We have added a cautionary note in the Discussion section and suggested that future studies with more diverse samples be conducted to further validate the distinctiveness of these constructs.

(5) We have carefully checked and revised the manuscript to ensure that it meets all PLOS ONE style requirements, including file naming, formatting of the main text, title, authors, affiliations, and references.

Best regards
